# Acoustic Assessment of Microstructural Deformation Mechanisms on a Cold Rolled Cu30Zn Brass

**DOI:** 10.3390/ma17133321

**Published:** 2024-07-04

**Authors:** María Sosa, Linton Carvajal, Vicente Salinas Barrera, Fernando Lund, Claudio Aguilar, Felipe Castro Cerda

**Affiliations:** 1 Departamento de Ingeniería Metalúrgica, Universidad de Santiago de Chile, Av. Ecuador 3735, Santiago 9170022, Chile; linton.carvajal@usach.cl (L.C.); felipe.castro@usach.cl (F.C.C.); 2 Grupo de Investigación Aplicada en Robótica e Industria 4.0, Instituto de Ciencias Aplicadas, Facultad de Ingeniería, Universidad Autónoma de Chile, Av. Pedro de Valdivia 641, Santiago 7500912, Chile; 3 Departamento de Física, Facultad de Ciencias Físicas y Matemáticas, Universidad de Chile, Avenida Blanco Encalada 2008, Santiago 8330015, Chile; flund@dfi.uchile.cl; 4 Departamento de Ingeniería Metalúrgica y Materiales, Universidad Técnica Federico Santa Maria, Av. España 1680, Valparaiso 2340000, Chile; claudio.aguilar@usm.cl

**Keywords:** brass, twinning, shear bands, nonlinear ultrasonic measurement, second harmonic generation, nonlinear parameter

## Abstract

The relationship between acoustic parameters and the microstructure of a Cu30Zn brass plate subjected to plastic deformation was evaluated. The plate, previously annealed at 550 °C for 30 min, was cold rolled to reductions ranging from 10% to 70%. Linear ultrasonic measurements were performed on each of the nine specimens, corresponding to the nine different reductions, using the pulse-echo method to record the times of flight of longitudinal waves along the thickness axis. Subsequently, acoustic measurements were conducted to determine the nonlinear parameter β through second harmonic generation. Microstructural analysis, carried out by X-ray diffraction, Vickers hardness testing, and optical microscopy, revealed an increase in deformation twins, reaching a maximum at 40% thickness reduction. At higher deformations, the microstructure showed the generation and proliferation of shear bands, coinciding with a decrease in the twinning structure and an increase in dislocation density. The longitudinal wave velocity exhibited a 0.9% decrease at 20% deformation, attributed to dislocations and initial twin formation, followed by a continuous increase up to 2% beyond this point, resulting from the combined effects of twinning and shear banding. The nonlinear parameter β displayed a notable maximum, approximately one order of magnitude greater than its original value, at 40% deformation. This peak correlates with a roughly tenfold increase in twinning fault probability at the same deformation level.

## 1. Introduction

Alpha brass (or yellow brass) is a copper–brass alloy with less than 35% zinc, where the zinc is dissolved to form a solid solution of uniform composition. The best-known alpha brass is Cu30Zn or cartridge brass, which has an optimum combination of properties, including a yield strength of 76 to 448 MPa, a tensile strength of 303 to 896 MPa, and high ductility with elongations up to 66%, making it suitable for cold work [1].

The principal mechanisms of plastic deformation in face-centered cubic (FCC) metals and alloys are slip via dislocation motion and deformation twinning. Cu30Zn has an FCC structure and a stacking fault energy of 14 mJ/m^2^ [2], which gives it the ability to form deformation twins and results in high strain hardening. Twinning breaks down a material’s microstructure into progressively smaller domains, an effect similar to grain refining, known as the dynamic Hall–Petch effect, which results in strengthening [3].

The critical stress needed for twinning is higher than for dislocation slip, and the nucleation of deformation twins also requires prior dislocation activity [4,5,6,7,8,9,10]. Studies [4,5] have shown that the onset of twinning in Cu30Zn during uniaxial tensile and compression tests is accompanied by a change in the slope of the strain hardening curve, that is, in a dσ/dε-type plot [11], an inflection point associated with critical twinning stress. During cold rolling reduction, it has been demonstrated [6,7,8,9,10] that twins are prominent features of the microstructure up to 40–50% reduction, while, at around 50-60% reduction, shear bands appear. These bands correspond to narrow zones of intense shearing strain that cut across grains.

Ever since the classic work of Fargette and Whitham [6] and Duggan et al. [10], it has been known that the microstructure of alpha brass, steels, and metallic materials, in general, undergoes increased complexity during large deformations [12]. This complexity has been the subject of extensive research. Recent examples of experimental studies include the examination of TWIP steels using optical microscopy, scanning electron microscopy (SEM), transmission electron microscopy (TEM), and electron backscatter diffraction (EBSD) at 75% deformation [13]. In another study, a high-entropy alloy was deformed up to a shear strain of 11% and analyzed using EBSD, TEM, X-ray diffraction (XRD), and plasticity simulations [14]. Similarly, a Ni-Ti alloy at 40% strain was studied using TEM, SEM, and XRD [15]. Commercial stainless steel underwent an 87% reduction in thickness, and this transformation was analyzed using electron microscopy and XRD [16]. Neutron diffraction and EBSD were utilized to study TiB_2_/Al composites with deformations of up to 95% thickness reduction [17].

The various microscopy techniques mentioned in the last paragraph, as well as neutron and X-ray diffraction, are probes that have been developed to a very high degree of sophistication. Nevertheless, they remain sensitive only to surface phenomena or are quite intrusive, as they do not probe the properties of the material itself but of a material that has been specially prepared to fit the requirements of a microscope, neutron reactor, or diffractometer. By contrast, ultrasound penetrates well into the bulk of a material and, due to the very low energies involved, is not intrusive. Because of these features, it lends itself well to the probing of pieces in service.

Linear or conventional ultrasonic techniques have been used as a nondestructive method for detecting defects such as cracks, pores, inclusions, and thickness loss [18,19,20,21]. These techniques, mainly associated with time of flight, echo detection, and attenuation measurement, are highly sensitive to millimeter- to micron-sized defects but less sensitive to features of a lower order of magnitude [22,23,24,25]. Although wave velocity measurement can be used to detect changes in microstructure associated with heat treatments or deformation [26], for microstructural features such as lattice distortion, crystallographic orientation, grain size variation, and microstructural defect density, wave distortion monitoring through nonlinear ultrasonic techniques provides a competitive material characterization technique [27,28,29,30,31,32,33,34,35,36,37,38,39].

A nonlinear acoustic experimental method that has been shown capable of detecting and monitoring microstructural changes is second harmonic generation (SHG) in metals [40]. In this method, a second harmonic wave is generated from the propagation and interaction of a monochromatic elastic wave with the medium. As the wave passes through the medium, it is distorted by microstructural features, resulting in harmonic generation. This nonlinear response is quantified through the nonlinear parameter β, which is formally defined in terms of linear combinations of second- and third-order elastic constants. However, since this parameter is part of the solution to the nonlinear wave equation, it is reduced to a proportionality factor between the amplitudes of the first and second harmonics, magnitudes that can be experimentally measured [40].

Phenomena such as slip dislocation, precipitation hardening, martensitic transformations, fatigue microcrack formation, and grain size are those whose nonlinear effect have been evaluated through harmonic generation. This harmonic generation arises, for example, from the movement and multiplication of dislocations due to the deviation of elasticity as plasticity arises [27,28,29,30]. During aging, the nonlinear effect is attributed to the matrix–precipitate coherence that develops due to the effect of heat treatment time and temperature [31,32]. In the study of fatigue microcrack formation, the nonlinearity is related to the formation of complex dislocation structures generating favorable places for microcrack nucleation [33,34]. The increase in nonlinearity due to the decrease in grain size can be attributed to the increase in grain-boundary area and its effect on waveform distortion [41].

In a recent work [42], dislocation density was monitored continuously in a standard tension test for 304L steel using ultrasound. Three consecutive loading–unloading cycles were performed, measuring the shear wave velocity, applied force, and sample strain, modeling the stress, strain, and sample thickness through finite elements. The resulting data for dislocation density and acoustic wave velocities as a function of stress in the first and third cycles agree with the predictions of Maurel et al.’s theory [43,44], which have been independently verified with results on aluminum [39,45], in which shear wave velocity showed a decrease at the onset of plasticity due to dislocation proliferation. The plastic behavior in the second cycle is markedly different, with an increase, rather than a decrease, in shear wave velocities. According to X-ray diffraction (XRD) measurements and a Croussard–Jaoul (CJ) analysis of the stress–strain curve, the change in wave velocity was attributed to the presence of an increasing number of twins in this intermediate cycle, and it was shown, using the model of Hirsekorn [46,47], that this wave velocity increase is consistent with a decrease in grain size due to a proliferation of twins.

Microstructural changes that take place in Cu30Zn brass during cold rolling are expected to cause disturbances in the propagation of ultrasonic waves. However, it is found that the wave interaction with twins has been little explored. The question arises as to how linear and nonlinear parameters respond to this deformation mechanism and how sensitive they are to the microstructural variations generated. Therefore, in this study, we present wave propagation velocities and the nonlinear parameter for Cu30Zn brass cold rolled up to 70% thickness reduction, relating the results to metallographic observations and results obtained in hardness and X-ray diffraction tests.

## 2. Materials and Methods

A Cu30Zn brass (UNS C26000) plate was homogenized at 550 °C for thirty minutes in a resistance furnace. Subsequently, cold rolling was carried out to obtain nine samples for thickness reductions of up to 70%, one for each applied deformation. The chemical composition and thickness are shown in Table 1 and Table 2, respectively.

### 2.1. Optical Microscopy and Hardness Test

**Figure 1:**
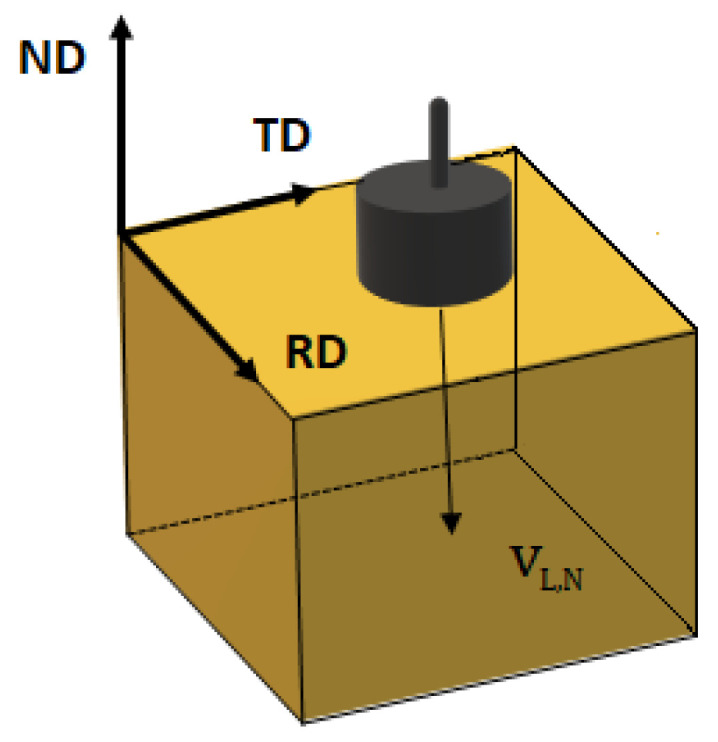
Longitudinal wave propagating along normal (ND) axis. (RD) and (TD) are rolling and transverse axes, respectively.

Metallographic analyses and hardness measurements were performed on the RD-ND plane (see Figure 1). Samples were prepared using standard grinding procedures, followed by polishing with 1, 0.3, and 0.05 μm alumina, and chemical polishing intervals with a copper polishing solution (50 mL acetic acid, 40 mL nitric acid, 10 mL orthophosphoric acid, and 1 mL hydrochloric acid). Finally, samples were polished with colloidal silica for 30 min and over-etched with a 5 gr FeCl_3_, 10 mL HCl, and 100 mL H_2_O solution.

Vickers hardness testing was conducted by a Zwick/Roell Vickers microhardness tester (Zwick Roell, Brierley Hill, UK). The hardness measurements were carried out under a 300 gf load, making ten indentations per sample.

Sidor et al. [48] have proven that the hardness test is a useful tool to estimate dislocation density. By approximating the flow stress $\sigma_{y}$ from the Vickers hardness $H_{V}$ ($\sigma_{y}$ = $H_{V}$/3.06), the stored energy $E_{D}$) caused by the presence of dislocations can be estimated from the measured $H_{V}$ values via the following relationship [49,50]:

(1) $$E_{D}=\frac{H_{V}^{2}}{G{\left[3.06M\alpha \right]}^{2}}$$

where *M* is the Taylor factor, α is the geometric constant, and *G* is the shear modulus. The stored energy and the dislocation density ρ are related to each other as follows [51]:

(2) $$E_{D}=\alpha \rho Gb^{2}$$

By combining Equations (1) and (2), one can estimate the dislocation density by using Equation (3):

(3) $$\rho_{H_{V}}=\frac{1}{\alpha^{3}}{\left[\frac{H_{V}}{3.06GM\alpha }\right]}^{2}$$

Although this is an approximate method, Sidor et al. [48] have obtained good results on aluminum samples when comparing the technique with the analysis of dislocations through XRD.

According to the texture analysis of Cu30Zn brass by Duggan et al. [10], initially, the Copper-texture component {211}〈111〉 remains almost constant, but then is largely depleted by between 40 and 60% thickness reduction, a range in which Brass {110}〈112〉 and Goss {110}〈001〉 texture are distinguished until a peak at 95% reduction is reached. Given the analysis mentioned above, the Taylor factors *M* used were those reported for Copper and Brass–Goss texture, 3.64 and 2.45, respectively [52]; the Burgers vector *b* = 0.26 nm [53], shear modulus *G* = 40 GPa [1], and α = 0.5 were employed for all deformations evaluated.

### 2.2. XRD Characterization

X-ray powder patterns of the samples were collected using a multipurpose powder diffractometer STOE STADI MP (STOE & Cie GmbH, Darmstadt, Germany), equipped with a DECTRIS MYTHEN 1K detector (DECTRIS AG, Baden, Switzerland). Pure Cu Kα radiation (λ = 1.54056 Å) was employed, with a curved Germanium (111) monochromator of the Johann type. XRD patterns were obtained between 40° and 100° in 2θ, with a step size of 0.10° and a holding time of 6 s per step.

The microstructural parameters were obtained using the Rietveld method, implemented with the Materials Analysis Using Diffraction (MAUD) software (Version: 2.999) [54,55]. LaB_6_ (with lattice parameter *a* = 4.1565915 Å) served as an external standard for determining instrumental broadening. The microstructure analysis involved profile fitting using the Delft line broadening model [56,57] and an anisotropic size–strain model based on Popa’s rule [58], harmonic texture [56,57], and consideration of planar defects (stacking faults and twins) using Warren’s model [59].

Cell parameter, microstrain, crystallite size, and twin fault probability (TFP) were extracted from the Rietveld refinements, with deviation calculated by varying the initial microstrain and crystallite size parameters for three refinements under the same conditions. Dislocation density was determined using the following formula [60]:

(4) $$\rho_{XRD}=\frac{2\sqrt{3}{\langle \varepsilon^{2}\rangle }^{1/2}}{bD}$$

where ${\langle \varepsilon^{2}\rangle }^{1/2}$ is the microstrain, *D* is the crystallite size (both values are directly obtained from MAUD post-refining), and *b* is the Burgers vector [61], which has been estimated to be 0.26 nm for FCC brass [53].

### 2.3. Acoustic Measurements

#### 2.3.1. Linear Acoustic Measurements

The 5 MHz central frequency longitudinal wave pulses generated by a Panametrics-5077PR pulse generator were induced normal to the RD-TD plane (see Figure 1) on each deformed specimen using the pulse-echo technique and a contact piezoelectric transducer (V406—Olympus longitudinal wave with element diameter 13 mm). The signals of the wave traveling along the thickness (the ND direction) of the samples were acquired by a Handyscope HS6 oscilloscope and analyzed by TiePie’s Multi-Channel software (Version 1.47.0). To determine longitudinal wave velocity, the time of flight was measured as the peak-to-peak time between the first two consecutive echoes. To obtain a standard deviation, measurements were taken at five different points on the RD-TD plane. As specified in Figure 1, $V_{L,N}$ is the longitudinal wave velocity, which is polarized along the propagation direction ND.

#### 2.3.2. Nonlinear Acoustic Measurements

**Figure 2:**
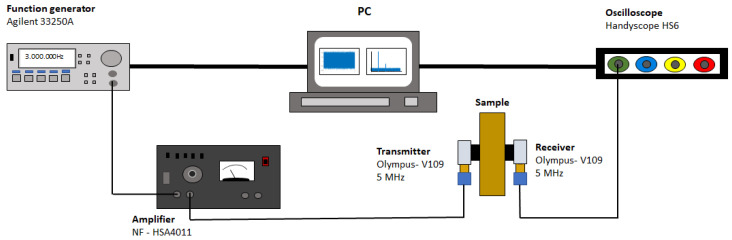
Schematic illustration of the experimental setup used for second harmonic generation.

Just as for the linear acoustic measurements, the plane of incidence for the fundamental and second harmonic voltage analyses of the deformed brass samples was the RD-TD plane, and the measurements were carried out following the experimental setup shown in Figure 2. Using an Agilent 33250A function generator (Agilent, Santa Clara, CA, USA) and an NF-HSA4011 amplifier (NF Corporation, Yokohama, Japan), a continuous longitudinal wave of frequency 3 MHz was transmitted into the material by a 12.7 mm element diameter Olympus—V109 transducer (Olympus, Toyko, Japan) (resonant at 5 MHz) placed on one side of the sample. The wave was received by an identical transducer and the signal was sent to a Handyscope HS6 oscilloscope (TiePie Engineering, Sneek, The Netherlands) connected to a personal computer. These procedures were implemented to ensure that the inherent nonlinearity of the measurement system, including the driving amplifier, piezoelectric transmitter, and acoustic coupling at the surfaces, was sufficiently suppressed. Then, through a Fourier analysis, the signal was transferred to the frequency domain, and the voltage of the fundamental harmonic ($V_{\omega }$) and the second harmonic ($V_{2\omega }$) was recorded. For data collection, an amplitude sweep was performed on the emitted wave, between 2.5 and 3 [V], and amplified by a factor of 10. The values of $V_{\omega }$ and $V_{2\omega }$ were obtained for each excitation amplitude. This procedure was executed for nine different points of each sample to test reproducibility and obtain scatter values.

The nonlinear parameter can be quantified by the following [40]:

(5) $$\beta =\frac{8A_{2\omega }}{x\kappa A_{\omega }^{2}}$$

where *x* is the elastic wave propagation distance [m], κ is the wave number [1/m], and $A_{\omega }$ and $A_{2\omega }$ are the absolute physical displacements of the fundamental and second harmonic waves [m]. Since reaching high accuracy on the calibration of the transducers is difficult and acquiring absolute displacements is quite complex, instead of calculating β in dimensionless form, $\beta^{*}$ is determined from the following:

(6) $$\beta^{*}=\frac{8V_{2\omega }}{x\kappa V_{\omega }^{2}}\left[m/V\right]$$

where $V_{\omega }$ and $V_{2\omega }$ are the amplitudes of the fundamental and second harmonics, measured in voltage units.

## 3. Results

### 3.1. Optical Microscopy and Hardness Results

**Figure 3:**
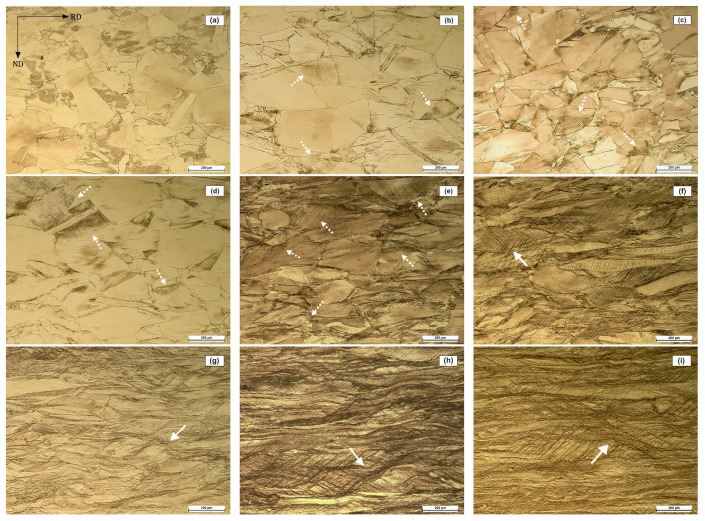
Microstructures of cold rolled CuZn30 brass at (**a**) 0%, (**b**) 10%, (**c**) 15%, (**d**) 20%, (**e**) 30%, (**f**) 40%, (**g**) 50%, (**h**) 60%, and (**i**) 70% deformation. The observation plane is the RD-ND plane, with the rolling direction along the horizontal axis of the micrographs. Dashed arrows indicate strain marks and solid arrows indicate shear bands.

Figure 3 illustrates the evolution of the microstructure with deformation. The observation plane is the RD-ND plane, with the rolling direction as the horizontal axis of the micrographs. Figure 3a shows the microstructure of the alpha brass in the softened condition (fully annealed), with a grain size of ≈45 μm, calculated by the planimetric procedure [62]. As deformation increases, grains become elongated along the rolling direction, and the number of strain marks increases, becoming visible at 10% thickness reduction (Figure 3b). These strain marks (indicated by dashed arrows) appear as groups of parallel lines within the grain, intersecting with other groups of lines. The micrographs also show the appearance of shear bands (indicated by solid arrows) after 40% deformation. These bands are identified as those following an angle between 35° and 45° to the rolling direction, associated with the main shear stresses [10].

**Figure 4:**
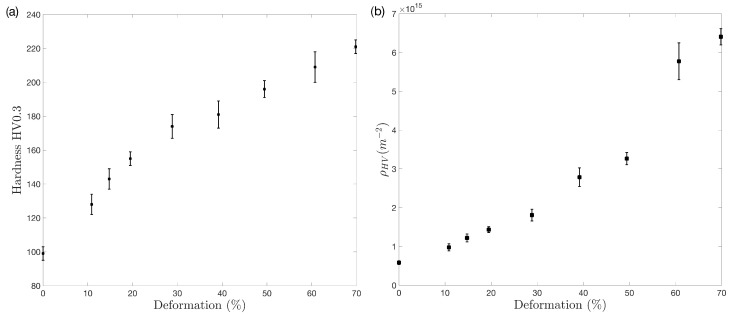
(**a**) Vickers hardness and (**b**) evolution of dislocation density calculated by the indentation method (Equation (3)) vs. deformation.

Figure 4a shows that, with increasing cold deformation, Vickers hardness exhibits a smooth rise characterized by two distinct changes in slope. Initially, the hardness increases with a relatively steep slope, which may be attributed to the rapid increase in dislocation density. However, beyond 30% deformation, the hardness curve undergoes a change in slope, becoming less steep. This indicates that, while the material’s hardness continues to increase, it does so at a slower rate, likely due to the activation of alternative deformation mechanisms.

Figure 4b depicts the evolution of dislocation density, calculated using Equation (3). Initially, there is a consistent and gradual increase in dislocation density as deformation rises. This early stage of deformation is marked by linear or nearly linear growth in dislocations, which aligns with the rise in Vickers hardness observed in Figure 4a. However, an intriguing shift occurs at 30% deformation. Beyond this point, the dislocation density experiences a much more significant increase compared to the initial stage. This pronounced rise in dislocation density suggests that the material might undergo a shift in its deformation mechanism or enter a new regime of plasticity.

### 3.2. XRD Results

**Figure 5:**
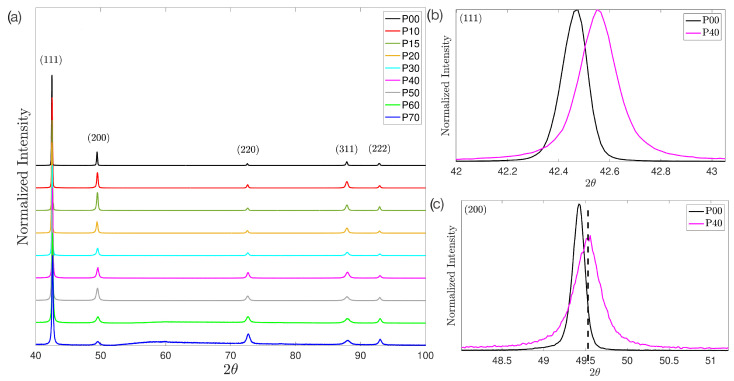
(**a**) XRD pattern of cold rolled Cu30Zn brass at different deformations. Five peaks are observed corresponding to different lattice planes: (111) (2θ = 42.46), (200) (2θ = 49.42), (220) (2θ = 72.54), (311) (2θ= 87.83), and (222) (2θ = 92.83). The curves go from top to bottom, increasing with the deformation. (**b**) Peak (111) for the softened (P00) and 40% deformation conditions (P40), showing displacement and broadening. (**c**) Peak (200) for the softened (P00) and 40% deformation conditions (P40). The dash line indicates displacement, broadening, and asymmetry.

Figure 5a shows the normalized XRD patterns for cold rolled Cu30Zn brass, displaying broadening, displacement, and asymmetry of diffraction peaks due to deformation. Displacement and broadening are shown in more detail in Figure 5b, where the peak (111) is shown for the softened and 40% deformation conditions. The above behavior is also observed for the peak (200) (Figure 5c), but in this case asymmetry stands out. It should be emphasized that displacement and asymmetry are a consequence of stacking faults and deformation twins; the competition between these sources of strain generated the variation in these peak aberrations [63].

Cell parameter, microstrain (${\langle \varepsilon^{2}\rangle }^{1/2}$), and crystallite size (*D*) are values provided by the MAUD program after the refinement process. Cell parameter was 3.682 ± 0.001 Å; microstrain and crystallite size are shown in Table 3, as well as the fitting parameter goodness of fit (GoF), and the weighted profile residual (Rwp), which indicate the fit of refinement processes. Microstrain constantly increases with a greater extent after 40% deformation; the crystallite size, on the other hand, decreases sharply from the initial annealing condition to 10% deformation and then decreases continuously to a lesser extent, which is consistent with the broadening of the X-ray diffraction peaks (Figure 5).

**Figure 6:**
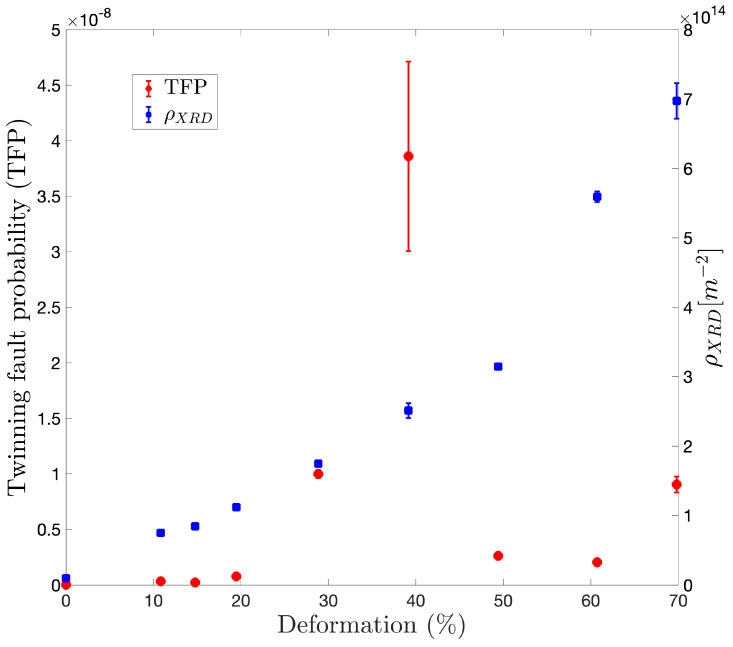
Twinning fault probability (TFP) (left axis) and dislocation density ($\rho_{XDR}$) (right axis) vs. deformation. Dislocation density was calculated according to Equation (4) and TFP was obtained directly from MAUD by considering the Warren’s Model.

Figure 6 shows twinning fault probability (TFP) on the left axis, obtained directly from MAUD by considering Warren’s Model [59]. TFP indicates the likelihood that a specific crystal will undergo twinning, and it implies that each successive layer (111) in the FCC sequence ABCABCABC should differ from the two preceding layers. Regions where the layers do not differ from the two preceding ones are identified as twin faults [59]. Notably, TFP becomes significant after surpassing 20% deformation, reaching its peak at 40%. Afterward, it gradually declines and rises once more at the highest strain level considered. On the right axis, the dislocation density is plotted, calculated according to Equation (4). It exhibits a continuous increase up to 40–50% deformation, followed by a steeper rise, signifying an increase in the dislocation density rate.

### 3.3. Ultrasound Results

**Figure 7:**
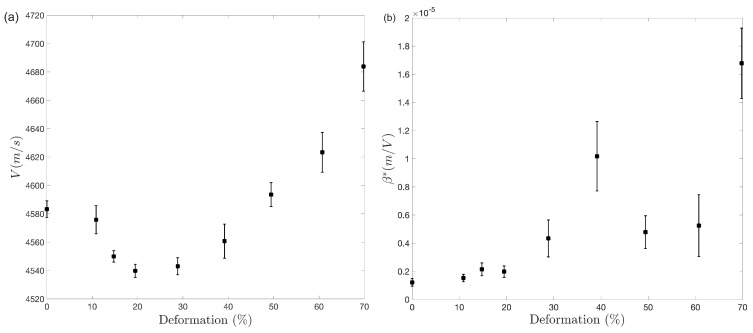
(**a**) Longitudinal wave velocity $V_{L,N}$ and (**b**) nonlinear parameter $\beta^{*}$ vs. deformation of cold rolled Cu30Zn brass.

Linear ultrasound results are displayed in Figure 7a as the velocities of the longitudinal wave ($V_{L,N}$), propagating along the thickness of the specimens. Their percentage changes with deformation are shown in Table 4. Figure 7a shows that $V_{L,N}$ reaches a minimum value of 4540 m/s, corresponding to 0.9% decrease, at 20% deformation, and subsequently a maximum of 4684 m/s (2.24% higher than the initial velocity) at 70% deformation. The nonlinear acoustic parameter $\beta^{*}$ is shown in Figure 7b, and its percentage change is in Table 4. $\beta^{*}$ reaches a first maximum value at 40% deformation, about 737% higher than in the annealed condition. $\beta^{*}$ subsequently decreases, remaining statistically constant in the 50–60% deformation range, and finally increases to 1280% at the highest strain evaluated. It is important to note that the large error bars of the nonlinear parameter are associated with the non-parallelism of the deformed specimens at the different measurement points and the high sensitivity of the parameter, generating some uncertainty, although not overlapping, between the different measurement points.

## 4. Discussion

Several authors [6,7,8,9,10] have reported an increase in strain marks with deformation in cold-deformed brasses. These marks may be attributed to slip lines and bands or clusters of primary deformation twins that increase with strain [10]. This finding is supported by the values obtained for TFP (Figure 6), which increase continuously from 10% to 40% deformation. After 40% deformation, the microstructure shows the presence of shear bands (white arrows in Figure 3g–i). The formation of shear bands at 40% deformation in cold rolled FCC metals has been reported elsewhere [64,65,66,67,68,69], described as narrow zones angled at about 35° to the RD direction. The appearance of these shear bands coincides with twinning saturation (TFP peak in Figure 6). Duggan et al. [10] explain that, at this stage, dislocation slip is inhibited by an array of closely spaced twin boundaries, and the only slip systems that can continue to operate are those for which the glide plane is parallel to the twin plane. Under these circumstances, the glide plane must rotate towards the rolling plane until the resolved shear stress is too small for further slip of this type. Consequently, the shear banding mechanism allows deformation to continue.

The observed formation of shear bands coincides with a drop in TFP. This observation could be explained by the detwinning process proposed by Hong et al. [70]. They describe the process of shear band formation as consisting of a nucleation state (bending, necking, and detwinning) and a thickening state. Nucleation of a shear band initiates in zones of localized deformation, bounded by a high density of dislocations resulting from strain accommodation. Necking of the twin lamellae starts at these boundaries by twin boundary (TB) migration. Continuous migration with deformation entails the breaking up and disappearing of some TBs, a process called detwinning.

Hong et al. [70] further describe that, by accumulating dislocations during the detwinning process, the subsequent shear strain can concentrate within shear bands, resulting in the formation of dislocation structures that evolve into a nano-sized sub-grain structure that is equiaxed or elongated along the shear direction. Other studies [71,72,73] have shown that one of the significant consequences of the development of these shear bands is their role in transforming the twin–matrix (T-M) lamellar structure to nano-sized grains at large strains. This assertion will be of interest in the analysis of the following results.

In Figure 4a, the hardness results show two slight changes in slope, each corresponding to specific deformation intervals. Figure 4b, representing the dislocation density curve, also exhibits two intervals with changes in slope, inversely related to the hardness curve’s slope changes. Up to 40–50% deformation, the dislocation density curve demonstrates a small slope, coinciding with the region where the hardness curve experiences its most significant hardening rate. This observation suggests that the material undergoes relatively sluggish dislocation multiplication during this stage, indicating that dislocation interactions might not be the main mechanism contributing to hardness. Instead, other mechanisms, such as twinning, could play a more crucial role in the observed hardening during this interval.

Once the material undergoes 40–50% deformation, the slope of the dislocation density curve increases. Simultaneously, there is a minor reduction in the hardness curve. These findings imply that, during this phase, the impact of twinning diminishes, and dislocation multiplication becomes the primary mechanism driving the hardening process.

The analysis of results obtained for dislocation density using XRD and Vickers hardness shows that hardness testing tends to overestimate XRD-based dislocation density by approximately one order of magnitude. This is to be expected since, at each deformation, a detailed analysis of the fraction of texture components is required in order to obtain a more accurate value of the Taylor factor M. The geometric factor α was estimated as 0.5, which has been described by the authors as overestimating the density of dislocations for low deformations. Likewise, hardness tests can cause the development of additional dislocations during the indentation process. However, it is to be noted that the trend of the XRD-based dislocation density curve is fairly well described by the hardness approach, which makes it of value as a first semi-quantitative approach.

As previously discussed, the hardness test enables a comprehensive assessment of crystal imperfections over a larger area (mm^2^) than XRD, which offers a more precise and detailed analysis of specific contributions like crystallite size (*D*) and deformation (${\langle \varepsilon^{2}\rangle }^{1/2}$) to dislocation density. The authors [48] have pointed out that using the indentation method to calculate dislocation density is an approximation, and certain factors need to be carefully taken into account to enhance the accuracy of such estimations. Nevertheless, despite its limitations, it serves as a valuable approach to illustrate the evolution of dislocation density in relation to hardness.

The change in velocity of the longitudinal wave $V_{L,N}$ (Figure 7a) indicates that cold rolling reduction in alpha brass can be broadly divided into two stages. The initial stage is characterized by the action of slip and twinning, known as crystallographic deformation mechanisms [4,5,6,7,8,9,10]. The decrease in wave velocity up to 20% deformation suggests that the effect of increasing dislocation density seems to prevail over twinning.

According to the theory developed by Maurel et al. [43,44], and verified with measurements on aluminum [45], steel, and copper [39,42], an increase in dislocation density generates a decrease in the wave velocity.

In the subsequent stage, the increase in wave velocity could be attributed to twinning and the formation of shear bands, both of which contribute to a refinement of the microstructure. This assertion finds further support in the theoretical propositions of Hirsekorn [46,47], who posits that a change in the mean grain size of a polycrystal of cubic symmetry leads to a change in the wave velocity. Evidence supporting the change in wave velocity due to the change in grain size is found in the literature [74]. Furthermore, in a recent study, Salinas et al. [42] observed a notable increase in wave velocity during tensile testing. They attributed this phenomenon to the emergence of twins within the material applying the model of Hirsekorn and demonstrating that an increase in wave velocity can be expected due to the reduction in grain size caused by twin proliferation. It is worth noting, however, that these works do not consider the potential influence of texture, which might also play a significant role in affecting wave velocity.

The influence of microstructural features on longitudinal wave velocity suggests that $\beta^{*}$ increases during the early stages of deformation due to slip dislocation and deformation twinning. However, XRD analysis data indicate that twinning significantly impacts the nonlinear parameter between 20 and 40% deformation. During this strain interval, there is a steady increase in $\rho_{XRD}$, while the substantial increase and saturation of twins likely contribute to a considerable rise in $\beta^{*}$.

The effect of decreasing grain size on the acoustic parameters has been explored under the assumption that grain boundaries act as crystallographic discontinuities, causing distortion in the ultrasonic wave propagation path. Mini et al. [41] investigated the effect of recrystallized grain size on the nonlinear parameter, reporting that an increase in nonlinearity can be attributed to the enlargement of grain-boundary and twin-boundary areas.

While the interfacial energy of a twin boundary is very small compared to that of an incoherent grain boundary, for wave propagation, a twin boundary can behave similarly to a grain boundary. The creation of a twin boundary increases the total energy of the system not due to a mismatch but to changes in bond angles [75]. Despite being a coherent boundary, the twin boundary still acts as a local stress in the material, possibly affecting wave propagation in a manner akin to grain boundaries.

The formation of shear bands is considered a non-crystallographic mechanism as it involves breaking the microstructure without following any crystallographic relation. From a microstructural perspective, this implies a shift in the material’s predominant deformation mechanism. The continuous increase in longitudinal wave velocity and decrease in the nonlinear parameter after 40% deformation could be attributed to shear band formation. While shear bands appear to affect longitudinal wave velocity similarly to deformation twinning, they exert a different impact on the nonlinear parameter. This complex evolution of nonlinearity indicates that it is becoming more sensitive to various microstructural phenomena than wave propagation velocity.

The microstructure within shear bands reportedly consists of nano-sized sub-grain structures [70,71,72], which, as discussed previously, can contribute to an increase in $\beta^{*}$. However, at this stage, the overall effect on acoustic parameters results from a combination of several factors, including the following:

- A substantial increase in the dislocation density.
- The destruction of the twinning structure, leading to the emergence of new sites for twinning nucleation with further deformation [70,71,72].
- The proliferation of shear bands.
- A significant component of Brass and Goss texture [10].

Each of these factors exerts a distinct influence on wave propagation when considered individually, but their collective interplay gives rise to a complex microstructure with balanced effects. This interplay contributes to observed changes in longitudinal wave velocity and the nonlinear parameter.

A comprehensive understanding of microstructural alterations and their influence on acoustic parameters is essential for gaining valuable insights into material mechanical characteristics. The intricate interplay among shear bands, dislocation nucleation, twinning structures, and texture underscores the complexity of the material’s response to deformation.

To fully grasp wave velocity and nonlinear parameter behavior in the context of cold rolling reduction in alpha brass, consideration of increased anisotropy and the development of crystallographic texture is crucial. These factors significantly contribute to the material response and must be carefully accounted for in analysis.

Our ongoing research integrates shear wave velocity measurements with detailed texture assessments to provide a more comprehensive understanding of the interplay between microstructural evolution, mechanical deformation, and wave velocity dynamics.

Exploration of increased anisotropy, crystallographic texture development, and integration of shear wave velocity measurements and texture assessments will be extensively pursued in future work. By incorporating these factors, we aim to offer a nuanced understanding of the complex behavior exhibited by materials undergoing cold rolling processes, contributing to the ongoing discourse in material science and engineering, and enriching the comprehension of wave propagation dynamics and its correlation with microstructural evolution.

## 5. Conclusions

The behavior of ultrasonic measurements in Cu30Zn brass has been correlated with the microstructure for deformation percentages up to 70%. Microstructural analysis revealed an increase in strain marks starting at 10% deformation and the formation of shear bands at 40% deformation. Diffraction analysis further suggested the presence of deformation twins, reaching a maximum at 40% deformation. Simultaneously, the calculated dislocation density exhibited a nearly linear increase during the early stages of deformation, followed by a significant rise after 40% deformation, coinciding with the formation of shear bands.

While XRD provides a more comprehensive and precise analysis of individual contributions to dislocation density, hardness testing serves as a good approximation to demonstrate the evolution of dislocation density with hardness. However, to improve the accuracy of dislocation density estimations, various factors such as the Taylor factor and the geometric factor alpha must be meticulously considered.

The increase in dislocations results in a 0.9% decrease in wave velocity at 20% strain. However, the combined effects of twinning and shear band formation lead to a significant increase in velocity, exceeding 2% at 70% deformation. Moreover, the simultaneous increase in dislocation and deformation twinning results in a remarkable rise of more than 700% in the nonlinear parameter *β* at 40% deformation. Considering the constant increase in dislocation density within this deformation range, the substantial increase and saturation of twins likely play a crucial role in the considerable rise of *β*.

Phenomena occurring after 40% deformation, such as a significant increase in the dislocation density rate, the reduction in the twinning structure, proliferation of shear bands, and the development of crystallographic texture, collectively interact to generate a complex microstructure. Within this intricate microstructure, the effects of these various factors balance each other, leading to significant changes in longitudinal wave velocity and the nonlinear parameter. Disentangling the effect of these various factors on the behavior of the longitudinal wave velocity and the nonlinear parameter at high deformation remains a challenging task.

## Figures and Tables

**Table 1:** CuZn30 brass chemical composition, wt% ^1^.

%Cu	%Zn	%Fe	%P	%Pb
69.1	30.76	0.026	0.029	<0.001
**%Mn**	**%Ni**	**%Si**	**%Cr**	**%Sn**
<0.0005	0.0021	<0.0005	0.0018	<0.0005

^1^ Measured by optical emission spectrometry using a SPECTROMAXx arc/spark metal analyzer.

**Table 2:** Thickness and deformation of cold rolled samples.

Sample	Deformation [%]	Thickness [mm] ^1^
P00	0.0	12.13 ± 0.01
P10	10.85	10.81 ± 0.01
P15	14.76	10.34 ± 0.02
P20	19.48	9.77 ± 0.02
P30	28.85	8.63 ± 0.01
P40	39.16	7.38 ± 0.01
P50	49.43	6.13 ± 0.01
P60	60.74	4.76 ± 0.02
P70	69.82	3.66 ± 0.03

^1^ Measured with Mitutoyo-Micrometer EXT. 0–25 mm (0.01 mm (103–137)).

**Table 3:** Microstructural characterization of the Cu30Zn cold rolled samples using the Rietveld Method.

	P00	P10	P15	P20	P30	P40	P50	P60	P70
**${\langle \varepsilon^{2}\rangle }^{1/2}$ × $10^{-3}$**	0.838	1.185	1.121	1.284	1.250	1.324	1.432	1.830	2.168
**${\langle \varepsilon^{2}\rangle }^{1/2}$ (error) × $10^{-5}$**	0.553	0.133	0.739	0.521	2.540	3.830	2.109	1.059	2.267
**D (Å) × ** $10^{3}$	11.488	2.105	1.774	1.529	0.950	0.694	0.607	0.459	0.042
**D (Å) (error) × ** $10^{1}$	77.040	3.919	1.968	1.572	1.059	0.964	0.717	3.212	0.602
**GofF**	2.40	1.68	1.57	1.96	1.32	1.86	1.25	1.72	1.22
**Rwp(%)**	14.72	13.24	12.17	13.83	10.74	14.75	10.11	13.41	8.14

**Table 4:** Percentage changes of linear and nonlinear acoustic parameters for cold rolled Cu30Zn specimens at different deformations.

	P00	P10	P15	P20	P30	P40	P50	P60	P70
**Δ*V_L_*_,_*_N_* (%)**	0	−0.16	−0.73	−0.95	−0.88	−0.49	0.22	0.87	2.19
Δβ* (%)	0	25.27	76.18	62.74	257.04	737.38	293.30	331.57	1280.62

## Data Availability

The original contributions presented in the study are included in the article, further inquiries can be directed to the corresponding authors.
